# A manifold-based approach to sparse global constraint satisfaction problems

**DOI:** 10.1007/s10898-019-00805-x

**Published:** 2019-07-12

**Authors:** Ali Baharev, Arnold Neumaier, Hermann Schichl

**Affiliations:** Faculty of Mathematics, University of Vienna, Oskar-Morgenstern-Platz 1, 1090 Vienna, Austria

**Keywords:** Decomposition methods, Diakoptics, Large-scale systems of equations, Numerical instability, Sparse matrices, Tearing

## Abstract

We consider square, sparse nonlinear systems of equations whose Jacobian is structurally nonsingular, with reasonable bound constraints on all variables. We propose an algorithm for finding good approximations to all well-separated solutions of such systems. We assume that the input system is ordered such that its Jacobian is in bordered block lower triangular form with small diagonal blocks and with small border width; this can be performed fully automatically with off-the-shelf decomposition methods. Five decades of numerical experience show that models of technical systems tend to decompose favorably in practice. Once the block decomposition is available, we reduce the task of solving the large nonlinear system of equations to that of solving a sequence of low-dimensional ones. The most serious weakness of this approach is well-known: It may suffer from severe numerical instability. The proposed method resolves this issue with the novel backsolve step. We study the effect of the decomposition on a sequence of challenging problems. Beyond a certain problem size, the computational effort of multistart (no decomposition) grows exponentially. In contrast, thanks to the decomposition, for the proposed method the computational effort grows only linearly with the problem size. It depends on the problem size and on the hyperparameter settings whether the decomposition and the more sophisticated algorithm pay off. Although there is no theoretical guarantee that all solutions will be found in the general case, increasing the so-called sample size hyperparameter improves the robustness of the proposed method.

## Introduction

### Aims

We consider square nonlinear systems

1 $$\begin{aligned} \begin{array}{l} F(x) = 0,\\ \underline{x} \le x \le \overline{x}, \end{array} \end{aligned}$$

where $F: \mathbb {R}^n \mapsto \mathbb {R}^n$ is a continuously differentiable vector-valued function, and whose Jacobian is structurally nonsingular; $\underline{x}$ and $\overline{x}$ denote the vector of lower and upper bounds, respectively on the components of *x*. The task we pose is to find a reasonably small set of points such that every solution of (1) is close to one of the points in this set. An algorithm solving this task finds in particular good approximations to all well-separated solutions. Even for problems with an infinite number of solutions, only a finite number of points need to be generated.

The task that we just posed is computationally intractable in general; we have to make further assumptions. We assume that (1) has already been ordered such that its Jacobian is in bordered block lower triangular form with small blocks and with small border width; the formal definition of bordered block lower triangular forms is given in Sect. 1.3. In Sect. 1.4 we give references how (1) can be ordered to the desired bordered block lower triangular form fully automatically and efficiently. We argue in Sect. 1.5 why the models of technical systems tend to decompose favorably in practice, and why the proposed method is expected to be useful across many engineering fields, e.g., mechanical, electrical, chemical, and aerospace engineering. Further (less limiting) assumptions are given in Sect. 1.6. The last one of our assumptions is given in Sect. 3, after the overview of the proposed method; this is necessary for better understanding of this particular assumption.

### Terminology

We refer to the number $\dim x$ of components of a vector *x* as its **dimension**. The **structural rank** of a matrix *A* is the maximum number of nonzero entries that can be permuted onto the diagonal with suitable row and column permutations. (It is also known as the maximal size of a transversal, of a maximum assignment, or of a maximum matching in the bipartite sparsity graph of *A*.) The structural rank is an upper bound on the numerical rank of *A*. *A* is nonsingular for some numerical values of its nonzero entries if and only if it is possible to permute the rows and columns of *A* in such a way that the diagonal is zero free. Such a matrix is called **structurally nonsingular**.

In an engineering application it is usually not meaningful to distinguish two solutions that are too close due to the intrinsic uncertainty of every real-life model. We therefore call a set *P* of points **well-separated** if, for any distinct points $p,q \in P$, the distance $\left\| p-q\right\| _2$ is above a small threshold $\delta$ specified by the user, for example $\delta =10^{-4}$.

**Array slicing notation.** The shorthand *p*:*q* is used for the ordered index set $p,p+1,\dots ,q$, where $p\le q$. When forming the subvector $v_{p:q}$ of a vector *v*, *p*:*q* is cropped appropriately if necessary; that is, invalid indices are ignored. The index set *p*:*q* is considered empty if $p>q$, and the expression $v_{p:q}$ is a valid subvector of *v* that has no components. In case of block vectors, the shorthand $v_{i:k}$ is used for a block vector with consecutive blocks $v_j$ ($j=i:k$).

A **point cloud** is a set of scattered points, intended to approximate a manifold.

### Bordered block lower triangular forms

**Fig. 1:**
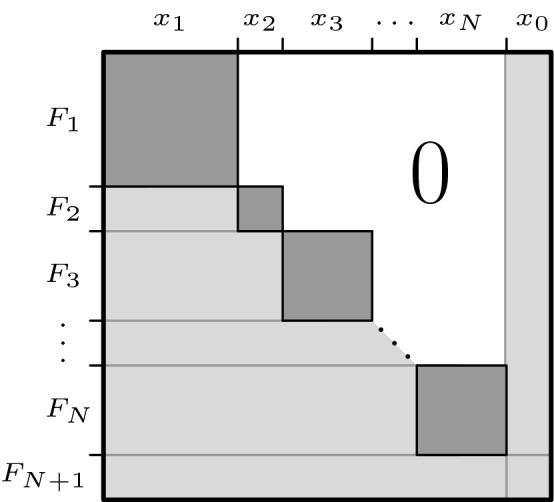
Bordered block lower triangular form with structurally nonsingular square blocks on the diagonal, see Eqs. (1)–(5). The square blocks along the diagonal (dark gray squares) must be structurally nonsingular. This decomposition can be computed fully automatically for any matrix that is structurally nonsingular, see Sect. 1.4. In engineering applications, the light gray area is typically sparse, and the border width and block sizes tend to be small, see Sect. 1.5

The so-called bordered block lower triangular form is illustrated in Fig. 1, and formally defined as follows. The variables in (1) are partitioned as

2 $$\begin{aligned} x = \left( \begin{array}{c} x_1 \\ \vdots \\ x_{N+1} \end{array} \right) \end{aligned}$$

into subvectors $x_i \in \mathbb {R}^{d_i} \quad (i=1\dots N+1)$, so that $n=d_1+\cdots +d_{N+1}$. For notational convenience, let

3 $$\begin{aligned} x_0:=x_{N+1}. \end{aligned}$$

Similarly to the variables, *F* is partitioned as

4 $$\begin{aligned} F(x) = \left( \begin{array}{c} F_1(x) \\ \vdots \\ F_{N+1}(x) \end{array} \right) \end{aligned}$$

into subfunctions $F_i(x) \in \mathbb {R}^{d_i} ~ (i=1\dots N+1)$. The Jacobian of the diagonal blocks, $F'_i(x_i)$ (see Fig. 1) are required to be structurally nonsingular.

For any bordered block lower triangular matrix, only variables from subvectors $$x_0, \dots , x_i$$$$(i\le N)$$ can appear in $$F_{i}(x)$$:

5 $$\begin{aligned} F_{i}(x) = F_{i}(x_0,~ x_1, \dots ,~ x_i)\quad \text{ for }\; i=1,\dots ,N. \end{aligned}$$

The motivation behind requiring a bordered block lower triangular form is that we can decompose the input system of equations (1) into a cycle-free sequence of subproblems, where the sequence is given by (5).

By construction, the diagonal blocks are structurally nonsingular. We refer to the set *S* of arguments where some block is singular as the **singular set** of the system. The structural nonsingularity implies that *S* has measure zero. For arguments *x* outside this set, all blocks are nonsingular, and $$F_{1:i}(x_{0:i})=0$$$$(i=1,\dots ,N)$$ implicitly defines a (possibly disconnected) $$d_0$$-dimensional manifold in $$\mathbb {R}^{d_i}$$, where $$d_i=\dim x_{0:i}$$. We refer to the full solution set of this subsystem for arguments within the original bounds as the **solution manifold** associated with the bordered block lower triangular form. (If the singular set *S* is nonempty, this is a manifold only in a generalized sense since it has singularities at the points of *S*, e.g. self-crossings and cusps.) In our algorithm we resolve this manifold through a coarse discretization by a point cloud.

Equations (2)–(5) describe the block sparsity pattern shown in Fig. 1. This decomposition exists for any structurally nonsingular matrix. As we will see in Sect. 3, the usefulness of a particular block decomposition depends primarily on the border width

6 $$\begin{aligned} d:=d_0=d_{N+1}, \end{aligned}$$

and secondarily on the largest block size

7 $$\begin{aligned} b:=\max {d_1,\ldots ,d_N}, \end{aligned}$$

from the point of view of the proposed method.

### Creating the desired block decomposition automatically

Sparse matrix ordering algorithms are a well-researched subject with a vast literature; we only mention some key points and references here. Both the Jacobian of (1) and the square blocks along the diagonal are required to have full structural row rank. The structural rank is revealed by the Dulmage–Mendelsohn decomposition (Dulmage and Mendelsohn [18–20], Johnson et al. [30], Duff et al. [17, Ch. 6], Pothen and Fan [43], and Davis [11, Ch. 7]). This decomposition is a standard procedure, and efficient computer implementations are available, for example HSL_MC79 from the HSL [29]. Practical ordering algorithms are applied next; these include the Hellerman–Rarick family of ordering algorithms [17, 21, 27, 28], and the algorithms of Stadtherr and Wood [45, 46]. An efficient computer implementation of the Hellerman–Rarick algorithms is MC33 from the HSL [29]. Although there are subtle differences among the various ordering algorithms, they all fit the same pattern when viewed from a high level of abstraction, see Fletcher and Hall [22].

### Tearing heuristics to create bordered block lower triangular forms

Beside the references given in Sect. 1.4, the engineering literature is also rich in sparse matrix ordering algorithms. Decomposing to bordered block lower triangular form has a long tradition in engineering applications: It is usually referred to as **tearing**, diakoptics, or sequential modular approach, depending on the engineering discipline. When dealing with distillation columns, tearing is called stage-to-stage or stage-by-stage calculations. Tearing dates back to the 1930’s [34, 48], and has been widely adapted across many engineering fields since: State-of-the-art steady-state and dynamic simulation environments all implement some variant of tearing, see for example Aspen Technology, Inc. [1], MOSAICmodeling [9], Dymola [10], JModelica [37], or OpenModelica [40]. The applicability of tearing is not limited to a particular engineering discipline: It is generic, and it is used in all state-of-the-art Modelica simulators to model “complex physical systems containing, e.g., mechanical, electrical, electronic, hydraulic, thermal, control, electric power or process-oriented subcomponents” [36].

The various tearing heuristics are concerned with selecting a minimal subset of variables called the torn variables; when these torn variables are moved to the border of the matrix, and the Dulmage–Mendelsohn decomposition is applied to the rest of the matrix, the blocks of the resulting bordered block lower triangular form correspond to the devices (or machines) of the technical system. The block sizes therefore tend to be *O*(1), that is, they are typically bounded by a small constant. More than five decades of practical experience and the wide-spread usage of tearing show that the tearing heuristics also tend to produce a narrow border when applied to technical systems.

### Further assumptions

Our algorithm assumes that the variables are adequately scaled. This allows us to use one of the standard norms to measure distances; unless otherwise indicated, we use the Euclidean norm ($\ell _2$-norm).

We also assume that the bound constraints $\underline{x} \le x \le \overline{x}$ are finite and reasonable; this is needed to allow an adequate sampling of the search space. Therefore, our method may not work well when a variable is unbounded or its upper bound is not known, and the user circumvents this by specifying a huge number such as $10^{20}$ as upper bound. Finite bound constraints are also important from an engineering perspective: These bounds often exclude those solutions of $F(x)=0$ that either have no physical meaning or lie outside the validity of the model.

## Overview of the proposed algorithm

The algorithm builds up a point cloud sequentially, satisfying

8 $$\begin{aligned} \begin{array}{l} F_{1:i}(x_{0:i}) \approx 0\quad \text{ for }\; i=1,\dots ,N,\\ \underline{x} \le x \le \overline{x}. \end{array} \end{aligned}$$

The algorithm starts with a scattered set of points $S^{(0)}$ for $x_0$, then eliminates the square blocks one-by-one along the diagonal in order $i=1,\dots ,N$, see Eq. (5) and Fig. 1. Solving (8) for $x_i$ will be referred to as **forward solve**.

If we applied forward solve only, the algorithm would be similar to Gaussian elimination without pivoting, which can give arbitrarily poor results even for well-conditioned linear problems [24, Ch. 3.3]. In the nonlinear case, and when propagating the point cloud within the variable bounds, the numerical issues manifest themselves in two ways:

- Many or all points become bound infeasible.
- The $x_i$ component of many points in the point cloud accumulate around one point or around a particular subspace. In this case, the remaining part of the feasible region is no longer adequately represented by the other points.

**Fig. 2:**
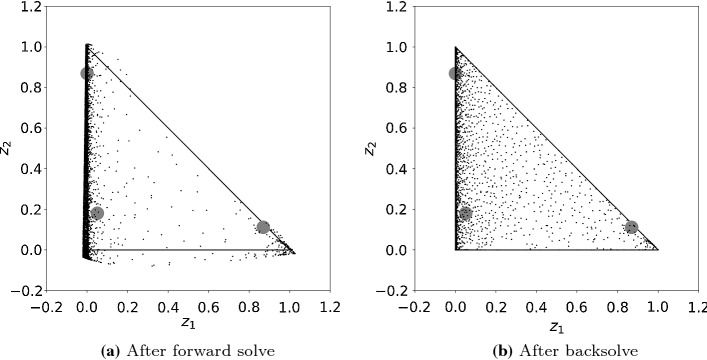
Illustrating how the backsolve step introduces new points on the test problem of Sect. 5 ($N=60$). **a** The scattered set of points after the forward solve in a particular iteration *i*, projected to 2D; $z_1, z_2$ are components of $x_i$; solid lines: boundaries of the feasible region, the so-called composition simplex. **b** The set of points after the backsolve step in the same iteration *i*. The three gray dots show the solutions

In both cases, the point cloud is no longer a proper approximation of the solution set of (8). Fig. 2a illustrates both issues on the test problem of Sect. 5: Some of the points are outside the feasible region (outside the so-called composition simplex), and many points have accumulated along the (0, 0)–(0, 1) line, while the interior part of the feasible region is poorly covered.

We propose the following procedure to mitigate the numerical issues. In each iteration step, after having solved (8) for $x_i$, new points are inserted for a subset of $x_i$ uniformly at random, let $(\tilde{x}_i)_J$ denote this subset where $\left| {J}\right| = d$ and *d* was defined at (6). (The $(\tilde{x}_i)_J$ denotes those components of the vector $\tilde{x}_i$ whose index is in the index set *J*.) Both the index set *J* and the values for $(\tilde{x}_i)_J$ come from a random number generator. We use the tilde to distinguish between $x_i$ and $\tilde{x}_i$: The former values come from the forward solve, the latter from a random number generator. These newly inserted $(\tilde{x}_i)_J$ points are still lacking the components of $x_{0:i}$ that are not covered by $(\tilde{x}_i)_J$. Therefore, for a given $(\tilde{x}_i)_J$, and for each point $x_{0:i-h-1}$ in the scattered set of points $S^{(i)}$, we solve the following NLPs:

9 $$\begin{aligned} \begin{aligned} \underset{{y}_{i-h:i}}{\text {minimize}}&\quad \left\| F_{i-h:i}(x_{0:i-h-1},y_{i-h:i})\right\| \\ \text {subject to}&\quad (y_i)_J = (\tilde{x}_i)_J \\&\quad \underline{x}_{i-h:i} \le y_{i-h:i} \le \overline{x}_{i-h:i} \\ \end{aligned} \end{aligned}$$

to determine the missing components, where *h* is a hyper-parameter of the algorithm (*h* stands for history, and it is typically a small integer). The missing components will partly come from the old point (the $x_{0:i-h-1}$ part), and the rest is the solution of the NLP above (the ${y}_{i-h:i}$ part); the $(\tilde{x}_i)_J$ part from the random number generator remains unchanged. The procedure of solving (9) will be referred to as **backsolve**. The new points are forcibly inserted in the backsolve step, it is therefore expected that there will be some amount of constraint violation in $F_{i-h:i}(x_{0:i-h-1},y_{i-h:i})$, which has to be tolerated. Figure 2 illustrates how the backsolve introduces new points.

The last subproblem at $i=N+1$ is different from (8) in that it is an overdetermined system, while all the other subproblems (8) are square. At $i=N+1$ the algorithm skips the forward solve step (since there is no square block to eliminate), and performs a backsolve-like step: It solves (9) with $J=\varnothing$, and with $x_{i-h:N}$ as starting points for $y_{i-h:N}$. (For the variables, the variable slices $i-h:i$ are truncated to $i-h:N$, see Sect. 1.2.)

The output of the proposed (main) algorithm, after finishing the last subproblem $i=N+1$, is a point cloud approximating the solution set of (1). The implementation details of the algorithm will be discussed in Sect. 4. The algorithm of the present paper is a significant improvement over older algorithms discussed in [3, 4], both algorithmically and on the implementation level. The entire algorithm has been redesigned and rewritten from scratch, and in particular, the backsolve step is radically different. Our numerical results show several orders of magnitude improvements in speed, while achieving better robustness at the same time.

## Exponential worst-case time complexity in the border width

As discussed at (8), the proposed method builds up a point cloud lying approximately on the implicitly defined solution manifold of

10 $$\begin{aligned} \begin{array}{l} F_{1:i}(x_{0:i})=0\quad \text{ for }\; i=1,\dots ,N,\\ \underline{x} \le x \le \overline{x}, \end{array} \end{aligned}$$

and aims at a point distribution such that every point on the manifold is close to a point of the cloud. (We refer back to Sect. 1.3 regarding the singular points.) For reasons of efficiency, the point cloud is constructed in a heuristic way, guided by theory.

Let *N*(*s*) denote the number of boxes intersecting the solution manifold when uniformly covering the bound constraint box by a grid of boxes of side $s>0$. We call $e:=\lim _{s\rightarrow 0}e(s)$, where $e(s):=\log N(s)/\log s$, the **effective dimension** of the solution manifold. As a consequence, the size of a cloud with the property that every point on the manifold has distance at most *s* to a point of the cloud grows for small *s* proportionally to $s^{e}$. In other words, constructing the point cloud will have a time complexity that is exponential in the effective dimension *e*. Creating the point cloud is therefore computationally tractable only for small effective dimensions *e*; how small depends on the resolution *s* needed, which fortunately is not high when (as usual) the total number of solutions of the original system is small, and the solutions are well-separated. Thus a small effective dimension *e* is the main assumption under which our new method can operate efficiently.

If the Jacobian of (10) has full rank then (since *d* equations are “missing” from the square system) the solution manifold has dimension *d*, the *d*-dimensional volume of the solution manifold is finite, and thus the effective dimension is $e=d$. But in general, pathological cases might be possible, such as Peano-like curves ($d=1$) that come close to every point in the box and then have large *e*. Excluding such pathological cases, which do not arise in most applications of interest, the border width *d* agrees with the effective dimension *e*.

In engineering applications, the presence of important bounds further decreases the effective dimension of the manifold. For example, we have the natural non-negativity bound on many variables. Each such bound will be active at many solutions, effectively amounting to an additional equation, typically decreasing the effective dimension by one. In addition, if the lower and upper bound on some variable differs by significantly less than the threshold *s*, this variable is effectively constant and also decreases the effective dimension. Such strong specifications are fairly common since the designer wants the system to perform something useful and therefore pushes the system to its limits (for example to create almost pure chemicals). We give numerical examples in Sects. 5 and 6 showing that the method is practical for certain difficult engineering applications.

To locate the solution manifold, i.e., to construct the approximating point cloud, we need to sample function values without knowing beforehand where the useful points lie that should go into the point cloud. To achieve this efficiently is the main reason why the bordered block triangular decomposition is needed. Indeed, we could sample the solution set of $F_{1:N}(x_{0:N})=0$ within the bound constraints directly, without decomposition. However, the volume to be sampled then grows exponentially with the dimension $p:=\dim {x_{0:i}}$, which gets larger and larger (ultimately $p=n$). This makes good sampling in this naive way prohibitively expensive. The proposed method avoids this scalability trap by sampling only at the square blocks along the diagonal (see Fig. 1): The volume to be sampled grows exponentially only with the largest block size, which is assumed to be reasonably small. In engineering applications, this assumption is usually satisfied since typically the largest block corresponds to the largest device/machine in the technical system being modeled.

## Implementation details of the proposed algorithm

A high-level overview of the algorithm was already given in Sect. 2. In this section we discuss the building blocks in more detail. These building blocks are mostly implementation-level details, and there could be other ways to fill-in these low-level details that the high-level overview left open.

### The source code of the algorithm

The most complete description of the algorithm is its source code, therefore the Python source code of the algorithm is available on GitHub [2] under the very permissive 3-Clause BSD License. For convenience the source code is distilled down to its essence, and it is given in “Appendix A” as pseudo-code too. Algorithm 1 of “Appendix A” is the core of the algorithm. We use the VA27 solver from HSL [29] to solve the equations and NLPs at each block. Since this solver cannot handle variable bounds, we enforce them with Algorithm 2. The backsolve step is given by Algorithm 3. The pseudo-code is less than 50 lines in total.

### The farthest-first subsampling algorithm

The goal of the subsampling algorithm is to select a spatially well-distributed subset of a given scattered set of points *S*. A greedy heuristic is implemented, based on the so-called farthest-first traversal. The algorithm starts by choosing a point in *S*. We currently pick the point closest to the mean of *S*; other choices are also possible, including the random choice. Then, points are selected one-by-one, always picking that not yet chosen point next that is the farthest away from the already chosen ones, breaking ties arbitrarily. The subsampling algorithm stops when the desired sample size is reached.

### Generating the new random points in the backsolve step

We refer back to Sect. 2, and to Fig. 2: After each forward solve we must insert new points into the sample where the manifold is not approximated properly. One way of populating such deserted areas would be inter- and extrapolation; this would assume that the spatial distribution of the points is already appropriate for inter- and extrapolation tasks, and assumes connectedness of the manifold. While this could be a viable approach, we chose a much simpler and more robust approach. Essentially we propose brute-force oversampling at the block level: We try to insert significantly more $(\tilde{x}_i)_J$ points than what we need. We do not know where to insert them, so we generate them uniformly at random within the variable bounds (brute-force). Then, the NLPs (9) of the backsolve step are solved, and those points whose objective (norm of the constraint violation) is above a user-defined threshold are discarded. Finally, we keep only the most distant ones of the remaining points by applying the subsampling algorithm.

This approach for populating deserted areas of the manifold is very robust, and fairly simple to implement. It does not assume connectedness, and it does not assume anything about the spatial distribution of the already existing points in the sample. In fact, if we loose all points in the forward solve, the backsolve may still succeed to insert new points, and the algorithm can continue. In contrast, it is impossible to inter- and extrapolate if we have lost all our points. Since we cannot assume connectedness of the manifold, some sort of (block-level) global sampling is inevitable.

### Efficient implementation of the backsolve step

A significant fraction of the execution time is spent in the backsolve step, solving (9). Three improvements proved to be crucial to perform the backsolve step efficiently: (i) trying only a small, carefully selected subset of all the possible combinations of the $((\tilde{x}_i)_J,~x_{0:i-h-1})$ matches in (9) instead of trying all of them, (ii) estimating a good starting point for (9), and (iii) skipping those matches that are very likely to have above-threshold objective value (constraint violation) at the optimum, and would most likely be discarded anyway.

As Sect. 2 is written, we try all the possible $((\tilde{x}_i)_J,~x_{0:i-h-1})$ matches in a brute-force manner. The previous implementation of the algorithm also worked [3] this way. Numerical evidence shows that it can be very wasteful: If two distinct points in the point cloud are close in their $x_{i-h:i}$ components, it is very likely that the $((\tilde{x}_i)_J,~x_{0:i-h-1})$ matches will have very similar objective value in (9) too; there is little to no benefit in trying both of them. An optional heuristic that we propose is to apply the subsampling algorithm of Sect. 4.2 to the points of the point cloud, considering their $x_{i-h:i}$ components only. We then try to match the points $(\tilde{x}_i)_J$ with this selected subset only. This heuristic can be disabled at the user’s discretion.

We propose estimating a starting point $y_{i-h:i}$ for (9) with singular-value decomposition (SVD, see [39, Ch. 10.2]). For simplicity, and since it seems to be adequate in practice, we currently ignore during this estimation the variable bounds in (9), and we also assume that a linear approximation to (9) around the optimum is appropriate. (This estimation is crude: We set parts of $\varDelta x_i$ to zero, although we let them change in (9) arbitrarily.)

**Fig. 3:**
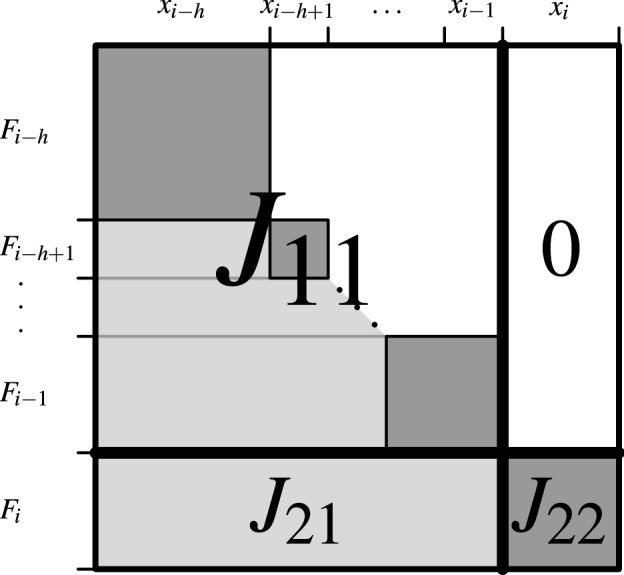
Submatrices $J_{11}$, $J_{21}$, and $J_{22}$ used in the starting point estimation for the backsolve step, see (11)

We consider the submatrices of the Jacobian *J* of *F*(*x*) shown in Fig. 3, and defined as follows. The rows of $J_{11}$ are the row blocks $i-h:i-1$ of *J*; those of $J_{21}$, and $J_{22}$ is row block *i* of *J*. The columns of $J_{11}$ and $J_{21}$ are the column blocks $i-h:i-1$ of *J*; those of $J_{22}$ is *i* column block *i* of *J*. The Jacobian is evaluated at $x_{0:i}$ (at the point $x_{0:i}$ that comes from the forward solve). In terms of these submatrices, the following linear least-squares problem is solved with SVD:

11 $$\begin{aligned} \underset{{\varDelta x}_{i-h:i-1}}{\text {minimize}} \left\| \left[ {\begin{array}{l} J_{11}(x_{i-h:i-1}) \\ J_{21}(x_{i-h:i-1}) \end{array}}\right] \left[ \varDelta x_{i-h:i-1} \right] - \left[ {\begin{array}{c} 0 \\ J_{22}(x_i)\varDelta x_i \end{array}}\right] \right\| _2^2 \end{aligned}$$

Informally speaking, (11) solves the linear approximation to (9) in which the variable bounds are ignored, $(\varDelta x_i)_J = (\tilde{x}_i)_J - (x_i)_J$, and all other components of $\varDelta x_i$ that are not in the index set *J* are set to zero. The solution to the linear least-squares problem (11) gives us $\varDelta x_{i-h:i}$, and our estimate for $y_{i-h:i}$ is $x_{i-h:i} + \varDelta x_{i-h:i}$.

The best match $$((\tilde{x}_i)_J,~x_{0:i-h-1})$$ for each $$(\tilde{x}_i)_J$$ is always tried. For those matches for which the norm of $$\left\| F_{i-h:i}(x_{0:i-h-1},y_{i-h:i})\right\| $$ at the starting point is below the pre-defined threshold (hyperparameter), we select at most $$m-1$$ additional candidate $$((\tilde{x}_i)_J$$, $$x_{0:i-h-1})$$ matches with subsampling. For each candidate match, we launch the local solver from the estimated $$y_{i-h:i}$$ to solve (9). The value of *m* is an arbitrary, used-defined value; in our numerical experiments $$m=20$$ was used, and we did not attempt to tune this hyperparameter.

## Numerical results: the effect of decomposition

We give numerical results where the computational gains, if any, are thanks to the block decomposition. The benchmark problems are coded in the AMPL modeling language [23], and are available on GitHub [2] together with the source code of the algorithm.

### Series of test problems

The steady-state simulation of distillation columns can be a major numerical challenge [13]. Our example is a series of challenging distillation columns; these columns have 3 solutions, one of which is missed even with problem-specific methods, see Sect. 5.2. Distillation columns consist of so-called stages. The natural order of the stages directly yields the desired block structure (2) and (4) by virtue of the internal physical layout of distillation columns; no preprocessing is necessary. (Even if it was not the case, we could use any of the ordering algorithms referenced in Sect. 1.4 and 1.5 to create the block structure fully automatically.) There is a one-to-one correspondence between the stages and the blocks.

In the engineering applications it is common to optimize the total cost by varying the number of stages, which makes distillation columns perfect test problems from the perspective of the present paper: Distillation columns have a natural parameter, namely the number of stages, for examining how different numerical methods scale as the number of blocks changes. As the number of blocks is varied (within reasonable limits) each column is interesting from an engineering point of view. Let *N* denote the number blocks. In our examples the size of each block is $4\times 4$ except the first block which is $2\times 2$; the problem size is 4*N*; the number of nonzeros is $25 N - 10$. The manifold dimension $d=2$, and it is independent of *N*.

The model equations are the MESH equations: The component material balance (M), vapor-liquid equilibrium (E), summation (S), and heat balance (H) equations are solved. The liquid phase activity coefficient is computed from the Wilson equations. The model and its parameters correspond to the Auto model [25], except for the number of stages *N* and the feed stage location $N_F$. The specifications are the feed composition (methanol–methyl butyrate–toluene), the reflux ratio, and the vapor flow rate.

There are three steady-state branches: two stable steady-state branches and an unstable branch; this was experimentally verified in an industrial pilot column operated at finite reflux [16, 25]. Multiple steady-states can be predicted by analyzing columns with infinite reflux and infinite length [5, 26, 42]. These predictions for infinite columns have relevant implications for columns of finite length operated at finite reflux.

### Numerical results published in the literature

The published numerical results for our test problem indicate numerical difficulties. Both the conventional inside-out procedure [8] and the simultaneous correction procedure [38] were reported to miss the unstable steady-state solution, see Vadapalli and Seader [49] and Kannan et al. [31] (all input variables specified; output multiplicity). However, all steady-state branches were computed either with the AUTO software package [12] or with an appropriate continuation method [25, 31, 49]. In both cases, the initial estimates were carefully chosen with the $\infty / \infty$ analysis [5, 26], and special attention was paid to the turning points and branch switching. Unfortunately, those papers do not include execution times, most likely because the computations involved human interactions too (initial estimates, turning points and branch switching).

### The baseline for comparisons

As discussed in Sect. 5.2, the literature clearly indicates that our benchmark problems are challenging, unfortunately the execution times are not available for comparisons; we have to establish a baseline for comparisons.

#### Requirements for the baseline algorithm

In order to assess the quality of our new method within the prior state of the art we need to compare against a suitable baseline method with similar capabilities. We use the following criteria that such a baseline method should possess. It should be

1. state-of-the-art;
2. able to enumerate all solutions of large, sparse systems;
3. able to handle transcendental equations and bound constraints,
4. usable from an advanced modeling language without user-input beyond equations and variable bounds;
5. a generic algorithm not tailored to a specific class of problems;
6. easy to use without any expert knowledge;
7. publicly available as an off-the-shelf solver.

To our knowledge, there is currently no such solver. But the technology to create one based on traditional techniques is available; so we wrote the baseline solver ourselves. We chose AMPL [23] as the modeling environment IPOPT [51] as local solver. Both are state-of-the-art, and their highly polished implementation is among the fastest ones. To enumerate all solutions, we implemented multistart with uniform random sampling between the variable bounds. (Uniform sampling is adequate since all variables are scaled to be between 0 and 1.)

#### Results with the baseline algorithm

IPOPT was executed from 250,000 randomly generated points for $N=50..74$, and 500,000 points were necessary for $N=75$ to get consistent results. Table 1 shows the relative frequencies of IPOPT finding a particular solution.

**Table 1:** Relative frequencies (percentages) of IPOPT finding a particular solution when starting points are generated uniformly at random between the variable bounds

*N*	Sol. 1	Sol. 2	Sol. 3	None
50	82.3	17.0	0.7	0.0
51	83.1	16.2	0.7	0.0
52	84.0	15.3	0.7	0.0
53	84.8	14.5	0.7	0.0
54	85.6	13.7	0.6	0.0
55	86.1	13.2	0.6	0.0
56	86.7	12.6	0.6	0.0
57	87.2	12.2	0.6	0.0
58	87.6	11.7	0.5	0.1
59	88.0	11.2	0.5	0.3
60	88.3	10.6	0.5	0.5
61	88.7	9.8	0.5	1.0
62	89.2	9.0	0.4	1.4
63	89.4	8.2	0.4	2.0
64	89.6	7.3	0.4	2.7
65	89.7	6.4	0.3	3.6
66	89.8	5.6	0.3	4.3
67	90.0	4.8	0.2	5.0
68	90.1	4.1	0.2	5.6
69	90.3	3.5	0.2	6.1
70	90.2	3.0	0.1	6.6
71	90.4	2.6	0.1	6.9
72	90.5	2.2	0.1	7.2
73	90.6	1.9	0.1	7.4
74	90.6	1.7	0.1	7.7
75	90.6	1.5	0.1	7.8

**Fig. 4:**
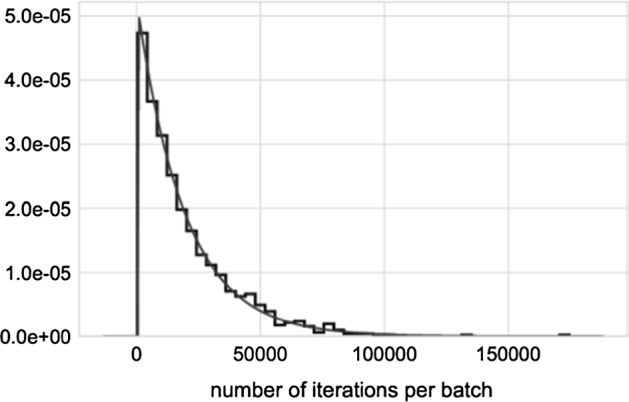
Histogram of the number of iterations per batch when the starting points are generated uniformly at random between the variable bounds; $N=60$. The counts are normalized to form a probability density, i.e., the area under the histogram will sum to 1. The fitted curve corresponds to the exponential distribution

**Fig. 5:**
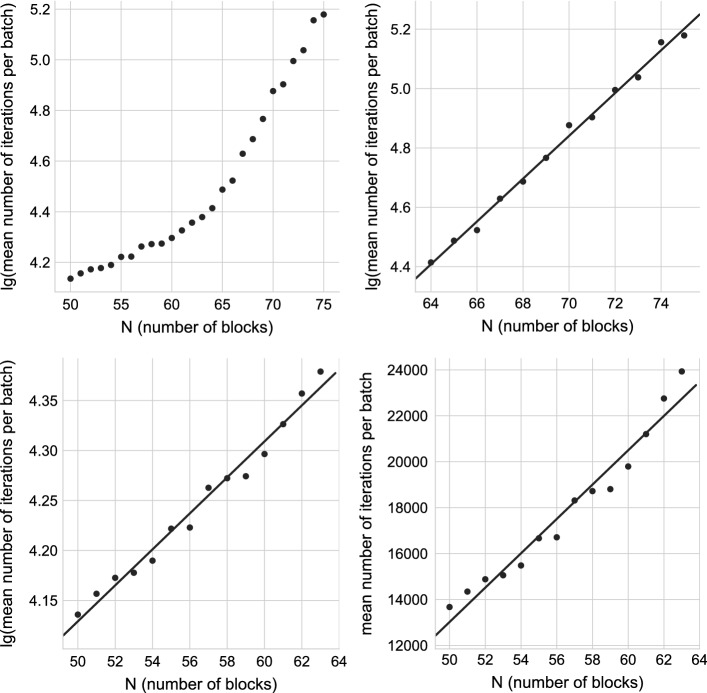
Computational effort of multistart with starting points generated uniformly at random between the variable bounds. The effort is measured as the mean number of iterations per batch, averaged over 250,000 starting points. The effort growth rate fits equally well with exponential and linear correlation in the $N=50..63$ regime, and it fits exponential growth rate between $N=64..75$

The points are partitioned into consecutive batches: The first batch starts with the first point. A batch is completed when all 3 solutions are found, and then the next batch starts. Only batches completed within the allocated point budget are considered (budget: 250,000 points for each $N=50..74$, and 500,000 points for $N=75$), that is, if the last batch is unfinished, we ignore it. For a fixed *N*, the total number of iterations per completed batch fits the exponential distribution, see Fig. 4 for $N=60$. The growth rate of the expected number of iterations in a batch fits equally well with exponential and linear correlation in the $N=50..63$ regime, and it fits exponential growth rate between $N=64..75$, see Fig 5. The total number of iterations IPOPT made in a batch correlates well with the total execution time, and with the number of starting points in the same batch.

### Results with the proposed method

#### Illustrating the point cloud computed with the proposed method

**Fig. 6:**
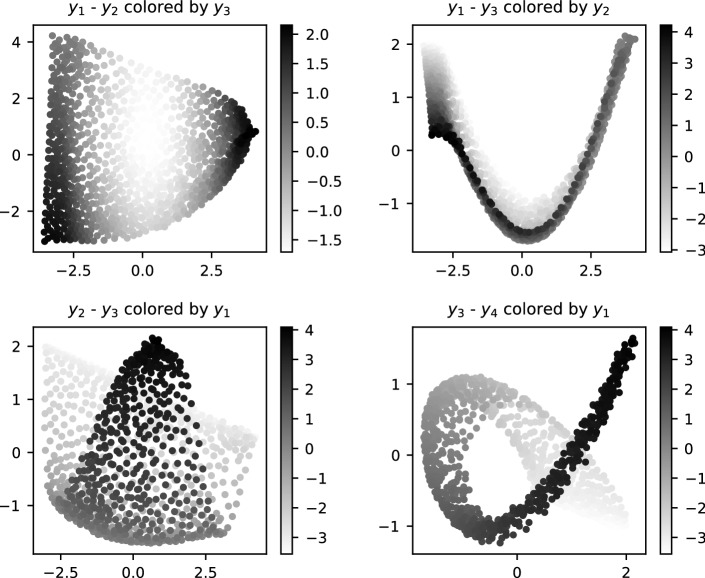
Representative sample of the 2D solution manifold of the leading subsystem $F_{0:30}(x)=0$, generated with the proposed method ($N=60$). The sample is projected to the planes of the principal components $y_i$–$y_j$

**Fig. 7:**
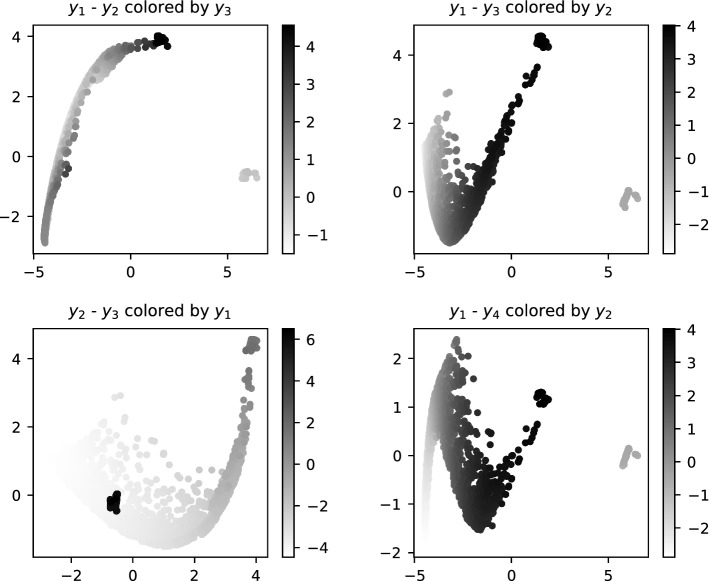
The starting points generated by the proposed method ($N=60$). The points are projected to the planes of the principal components $y_i$–$y_j$. There would be 3 well-separated clusters of points in the ideal case, however, the last two equations $F_{N+1}(x)=0$ only trim the 2D solution manifold of the leading subsystem $F_{0:N}(x)=0$ due to mild ill-conditioning. One cluster is nevertheless fairly small and well-separated

Figure 6 shows the intermediate point cloud in iteration $i=30$ for $N=60$, projected to 2D with principal component analysis (PCA). We used Scikit-learn [41] to perform PCA and to generate the plots. Fig. 7 shows the final output of the proposed method, the generated starting points, projected to 2D with PCA. Higher principal components produce (as to be expected) more wiggly projectors that do not represent true features of the point cloud.

#### Illustrating the point cloud with manifold learning

We also investigated the manifold structure using manifold learning. We tried each manifold learning algorithm available in Scikit-learn: Isomap [47], locally linear embedding (LLE) [44], modified locally linear embedding (MLLE) [53], Hessian Eigenmapping (also known as Hessian-based LLE or HLLE) [15], Spectral Embedding (Laplacian Eigenmaps) [6], local tangent space alignment (LTSA) [52], and multidimensional scaling (MDS) [7, 32, 33]. (Although the t-distributed Stochastic Neighbor Embedding [35, 50] algorithm, or t-SNE, also proved to be robust, we did not use t-SNE for the present paper: It was designed to artificially exaggerate structure in the data to reveal clusters, but that is undesirable in our case.)

It is not uncommon that the embeddings show false structures that in reality are not present in the data. Problem-specific knowledge was used to recognize any false structure in the embeddings as follows. We colored each point: The mole fractions of the 3 chemical components in the liquid phase on stage *k* of the distillation column are chosen as the coordinates in the RGB color space, where the stage index *k* is a parameter. In short, the color of the point corresponds to the chemical composition of the liquid phase on stage *k*. The theory of distillation – in particular the so-called residue curve map [14] of the mixture – tells us that for a fixed *k* we should see a smooth color transition in the embeddings, similar to the smooth shade transition in Fig. 6. Furthermore, the coloring of the points in the embeddings should change only smoothly when *k* is increased or decreased by 1.

We inspected these color transitions for each algorithm offered by Scikit-learn. If the manifold learning algorithm creates a wrong embedding or false structure, it is obvious at first glance. In our numerical experience, among the manifold learning algorithms implemented in Scikit-learn, only multidimensional scaling was robust enough to consistently produce correct embeddings without any hyperparameter tuning. A possible explanation for its robustness could be that it randomly chooses the initial configuration; the other embedding techniques that we listed are based on a nearest-neighbor search which can be fooled if the points happen to have unfortunate distribution in the original high-dimensional space. The downside of multidimensional scaling is that it was by far the most computationally expensive manifold learning algorithm of all tried. Note, however, that multidimensional scaling is not part of the proposed method; it is used only for visualization.

**Fig. 8:**
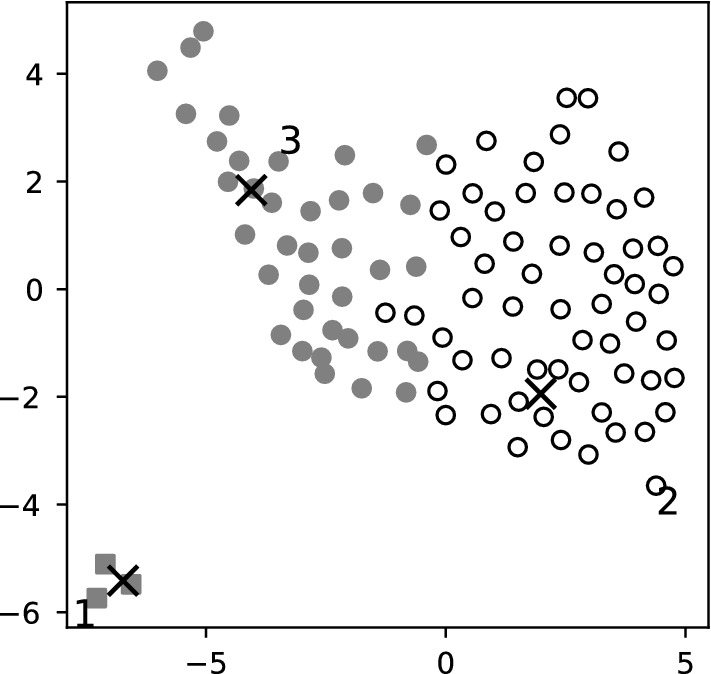
The starting points (circles and squares) generated by the proposed method ($N=60$), embedded into the 2D with plane with multidimensional scaling. The 3 crosses show the 3 solutions. Each cluster of starting points yields the solution it surrounds when the IPOPT solver is started from there. The first 3 points picked by the farthest-first heuristic of Sect. 4.2 are marked with 1, 2, 3; in this case, they suffice to find all solutions. Note that the farthest-first heuristic measures distances in the original high-dimensional space

Multidimensional scaling seeks a low-dimensional representation of the data in which the distances sensibly approximate the distances in the original high-dimensional space (the between-points distances are preserved as well as possible). Using multidimensional scaling from Scikit-learn, we unfolded the discretized 2D manifold formed by the generated starting points into the 2D plane and found the embedding shown in Fig. 8.

#### Running a local solver from the output of the proposed algorithm

The subsampling algorithm of Sect. 4.2 selects the points in a specific order; the subsampling procedure can be used to order the points in any set *S*. This order is the so-called greedy permutation or the farthest-first traversal. When the main algorithm finishes, we propose that a local solver for large-scale, sparse problems (like IPOPT) is launched from the points of the final point cloud in this order. The numerical experiments suggest that this increases the likelihood of finding all solutions early, because we always try that point next that is the least similar to the already tried ones. As it is shown in Fig. 8, the first 3 points picked by the farthest-first heuristic suffice to find all solutions in this case. Note that in Sect. 5.3 the probability of finding the third solution was $0.5\%$ for starting points generated uniformly at random between the variable bounds; see in Table 1, row $N=60$.

Numerical experiments also show that the final constraint violations are non-distinctive with respect to the goodness of the starting points: Below a certain threshold, the constraint violations are due to the random perturbations applied in the backsolve step, and they do not convey any information regarding the goodness of the starting points. In other words, the constraint violation is not a good metric for ordering the final starting points; we propose the farthest-first traversal instead.

### Comparisons: The effect of decomposition

**Fig. 9:**
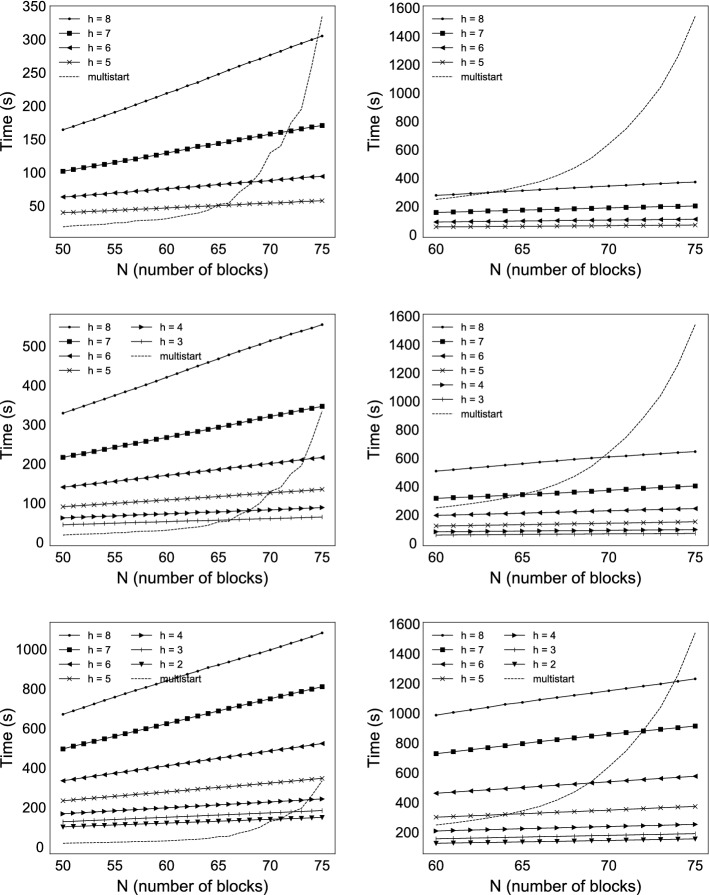
Comparing the execution times of the proposed method to multistart with randomly generated starting points between the variable bounds. For multistart, the mean execution time for a batch is given, averaged over 250,000 starting points. The execution times for the proposed method are all-inclusive: IPOPT is launched from the first 6 points picked by the farthest-first heuristic which suffices to find all 3 solutions. Left side: All solutions are required for the given column lengths. Right side: All solutions to 10 different columns with consecutive length are required; the execution times are plotted at the longest column. Rows from top to bottom: $M_{keep}=100$, 200, 400; for the meaning of the algorithmic parameters *h* (history), $M_{keep}$ (at most this many new points are inserted in each iteration) see the pseudo-code in “Appendix A”

The effect of the decomposition (2)–(5) can be studied by requesting all solutions for a given column length, and comparing the execution times of the proposed method with the baseline multistart algorithm (no decomposition). As we discussed in Sect. 5.3, if the starting points are generated uniformly at random within the variable bounds, the computational efforts grow exponentially for $N\ge 64$. For the proposed method, the computational efforts grow linearly, thanks to the decomposition. It depends on the problem size (column length) and on the hyperparameter settings whether the decomposition, and the more sophisticated algorithm pays off; see the left column of Fig. 9, comparing the execution times.

## Numerical results: reusing shared substructure

A frequent task in engineering is to solve a series of related square systems $F^{\ell }(x)=0$, where the number $N_\ell$ of blocks of the $\ell$th problem and hence the Jacobian varies, but the equations in the first $B_\ell$ blocks of $F^{\ell }$ and $F^{\ell +1}$ are identical; the remainder may deviate arbitrarily. If $B_\ell$ is close to $N_\ell$, the major part of the point cloud can be reused without any change.

We give numerical results where the computational gains, if any, are thanks to the reused substructure. The benchmark problems and the baseline algorithm are the same as in Sect. 5. The difference is that all solutions to 10 different columns with consecutive length are required. The shared substructure can be reused with the proposed method. This results in significant gains compared to our baseline multistart method, see the right column of Fig. 9. As previously, it depends on the problem size, and on the hyperparameter settings whether the decomposition, and the more sophisticated algorithm pays off.

## Future work

*Nonlinear programming with optionally varyingNand*$\ell$ Another common application in the field of engineering is to augment the leading subsystem of $F_{1:N}(x_{0:N})=0$ of (1) with an objective function and ask for all global optima.

12 $$\begin{aligned} \begin{array}{ll} \min &{} G^{(N,\ell )}(x_{0:N}) \\ \text{ s.t. } &{} F_{1:N}(x_{0:N}) = 0 \\ &{} \underline{x} \le x \le \overline{x} \end{array} \end{aligned}$$

The basic algorithm, sketched in Sect. 2, is applied to $F_{1:N}(x_{0:N})=0$ up until and including block *N* as before to obtain a point cloud, approximately satisfying the constraints of (12). Then, a local solver is executed from the points of the point cloud, targeting the nonlinear program (12). As for the computational savings with varying *N* and $\ell$, the same arguments hold as in the previous paragraphs: Whether the leading underdetermined subsystem is augmented with *d* additional equations (making it square), or with an objective function, the point cloud for the shared leading subsystem $F_{1:N}(x_{0:N})=0$ can be reused either way.

Chemical engineering use cases include the following scenarios. The steady-state model of a distillation column is given without the reflux specification, that is, the system of equations is underdetermined by one degree of freedom. First, we compute a point cloud, approximately satisfying this system, with Alg. 1 halting at line 10. Then, the engineer can specify

- the reflux ratio, that is, provide one more equation, making the system square;
- or the reflux molar flow rate, making the system square;
- or append an objective function, and look for the cost optimal reflux ratio.

In each case, the engineer can (re)use the point cloud for free. There is still some additional computational work left due to the newly added equation or objective function, but that work is negligible compared to computing the point cloud. We expect significant computational benefits where a precomputed point cloud can be reused several times at no additional cost thanks to shared substructure.
